# TCF-1 deficiency influences the composition of intestinal microbiota and enhances susceptibility to colonic inflammation

**DOI:** 10.1007/s13238-020-00689-8

**Published:** 2020-01-22

**Authors:** Guotao Yu, Fang Wang, Menghao You, Tiansong Xu, Chunlei Shao, Yuning Liu, Ruiqi Liu, Min Deng, Zhihong Qi, Zhao Wang, Jingjing Liu, Yingpeng Yao, Jingjing Chen, Zhen Sun, Shanshan Hao, Wenhui Guo, Tianyan Zhao, Zhengquan Yu, Qian Zhang, Yaofeng Zhao, Feng Chen, Shuyang Yu

**Affiliations:** 1 State Key Laboratory of Agrobiotechnology and Beijing Advanced Innovation Center for Food Nutrition and Human Health, College of Biological Sciences, China Agricultural University, Beijing, 100193 China; 2 Central Laboratory, School of Stomatology, Peking University, Beijing, 100081 China

**Dear Editor,**

The diversity and composition of gut microbiota play critical roles for maintaining the homeostasis of host commensal bacteria which are correlated with mammalian health (Ding and Schloss, 2014). Disorder of microbial community is implicated in the pathogenesis of several human diseases, such as autoimmune disease, allergy, diabetes, obesity, and inflammatory bowel disease (IBD), which is clearly demonstrated to relate to gut dysbiosis in patients and mouse models (Saleh and Elson, 2011).

The dynamic cross-talk between immune genes and host commensal bacteria and the mechanisms of host-microbiota mutualism have captivated widely research interests in the past few years. Gut-associated lymphoid tissues (GALTs), including various immune cells around intestinal mucosa, the Peyer’s patches (PPs) and the mesenteric lymph nodes (MLNs), are critical for intestinal immunity. Accumulating studies have shown that immune genes influence homeostasis of the microbiota composition through innate and adaptive immune arms correlated with deficient GALTs. Toll-like receptor 5 was critical for colonizing flagellated bacteria by mediating REG3γ production (Fulde et al, 2018). Loss of innate adaptor MyD88 impaired T follicular helper (Tfh) cells and IgA-producing B cells development, resulting in failure to control the bacterial community due to defects of germinal center response (Kubinak et al, 2015). The inhibitory co-receptor programmed cell death-1 (PD-1) deficiency resulted in impaired IgA-producing B cells in the germinal center of PPs and the imbalanced composition of microbial community (Kawamoto et al, 2012). Together, defects of immune regulatory genes in GALTs lead to impaired germinal center response and reduced IgA expression level, which are related to the imbalance of the gut bacterial community (Kato et al, 2014).

The transcription factor TCF-1 (encoded by *Tcf7*) is well known to play essential roles in different developmental stages of T, NK cells, and ILCs (Held et al, 2003; Mielke et al, 2013; Verbeek et al, 1995). These immune subsets are essential for establishing GALTs and maintaining the balance of gut associated immunity. Emerging studies have shown that TCF-1 is also critical in regulating differentiation of Tfh cells (Xu et al, 2015), which is a key T cell population to help germinal center B cell producing antibodies. Although these important functions of TCF-1 have been well elucidated, the relationship between TCF-1 and gut bacterial composition remains unclear. In this study, we aim to decipher the role of TCF-1 involved in the homeostasis of gut microbial community and inflammation.

In view of the importance of TCF-1 for GALTs, we first examined MLNs and PPs in TCF-1 deficient mice (*Tcf7*^−/−^ mice) and littermate controls. Similar to previous reports, *Tcf7*^−/−^ mice showed invisible PPs due to abnormal development (Fig. 1A) (Harly et al, 2019; Mielke et al, 2013; Yang et al, 2015), and smaller size of MLNs with diminished cell numbers (Fig. 1B). We next focused on IgA-producing B cell subsets in MLNs. The frequency and cell numbers of PNA^+^Fas^+^ germinal center B cells (GCBs) in TCF-1 deficient MLNs were dramatically diminished (Fig. S1). Although the frequency of CD138^+^CD19^lo^ plasma cells (PCs) was not statistically significant (Fig. S1C) in *Tcf7*^−/−^ mice, the cell numbers were notably decreased (Fig. S1D). The IgA level in fecal supernatants was reduced dramatically in *Tcf7*^−/−^ mice (Fig. 1C). Correspondingly, an obvious reduction of IgA-binding microbiota of intestinal contents from *Tcf7*^−/−^ mice was detected (Fig. 1D). These data indicated that ablation of TCF-1 leads to diminished GCBs and PCs in MLNs and the low level of IgA, which are tightly correlated with the reduction of IgA-binding bacteria in gut.

**Figure 1:**
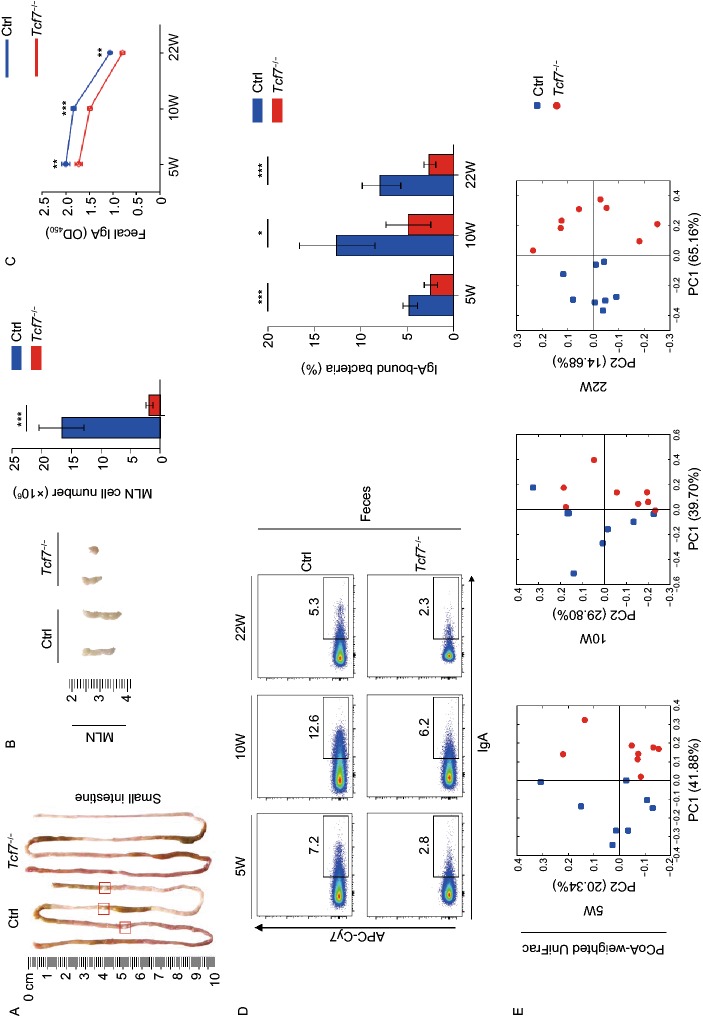

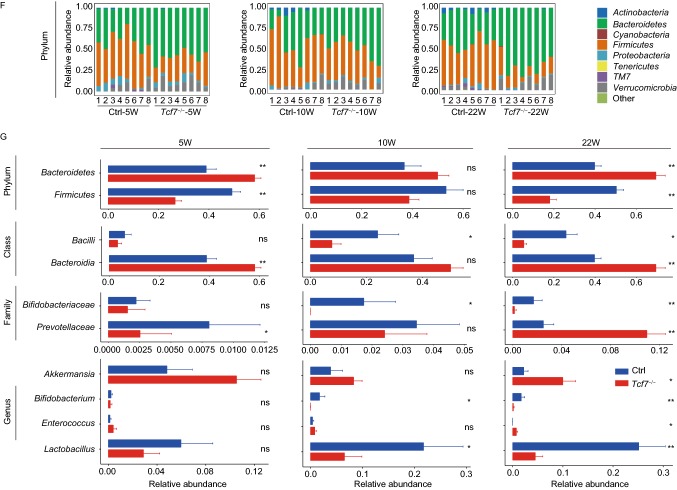
**TCF-1 deficiency impairs IgA secretion and leads to dysbiosis in gut microbial community**. (A) No visible Peyer’s patches (PPs) were observed in *Tcf7*^−/−^ mice (*n* ≥ 4 for each group). The size and numbers (B) of MLNs from indicated genotypes were shown (*n* ≥ 4 for each group). IgA levels of feces (C) from indicated genotypes were measured by ELISA (*n* ≥ 5 for each group). (D) IgA-binding bacteria in feces were analyzed by flow cytometry. Frequency of IgA-binding bacteria in feces was shown statistically (*n* ≥ 6 for each group). Data are mean ± SD and representative of at least two independent experiments. *P < 0.05, **P < 0.01, ***P < 0.001 (Student’s *t*-test). (E) PCoA plot of weighted UniFrac distances showed separation of microbial community by genotype within co-housed animals (*n* = 8 for each group) at 5, 10 and 22 weeks old. Each point represents a gut microbial community from an individual mouse. (F) Relative bacterial abundances (*n* = 8 for each group) were shown at the level of phylum. (G) Representative bacteria at phylum, class, family and genus abundances in feces were shown at 5, 10 and 22 weeks old. Significant differences were determined by Wilcoxon test. *P < 0.05, **P < 0.01, ***P < 0.001

To determine whether loss of TCF-1 impaired the production of IgA during bacteria invasion, we detected the germinal center response using *Listeria* infection model. On day 7 after infection, Tfh and GCB cells were analyzed from *Tcf7*^−/−^ mice together with littermate controls. We found the frequency and numbers of SLAM^lo^CXCR5^+^ Tfh cells were significantly decreased in SPs and MLNs from *Tcf7*^−/−^ mice (Fig. S2A). PD-1^hi^CXCR5^+^ GC Tfh cells in *Tcf7*^−/−^ mice also exhibited a significant reduction (Fig. S2B). Meanwhile, PNA^+^Fas^+^ GCBs in SPs and MLNs from *Tcf7*^−/−^ mice were remarkably diminished in response to *actA*^−^LM-Ova challenge (Fig. S2C). Accordingly, the frequency and numbers of CD138^+^CD19^lo^ PCs in SPs and MLNs were also much lower from *Tcf7*^−/−^ mice than controls (Fig. S2D). Impaired PNA^+^ GCs within B cell follicles of SPs and MLNs from *Tcf7*^−/−^ mice were further confirmed by immunohistochemical analysis (Fig. S2E). As a result, the levels of antigen specific IgA and IgG in feces were significantly decreased in absence of TCF-1 (Fig. S2F). Collectively, these results indicated that TCF-1 is essential for Tfh cells differentiation as well as germinal center responses to produce antigen specific IgA upon acute bacterial infection.

Given age as a key factor for establishing steady composition of intestinal bacteria, mice at 5, 10 and 22 weeks old were selected to perform the analysis of 16S ribosomal RNA (rRNA) sequencing. We found that the microbial communities of *Tcf7*^−/−^ mice at different ages were all notably distinct from their littermate controls by the principal coordinates analysis (PCoA) on the weighted Unifrac distances (Fig. 1E). Alpha diversities were calculated with several metrics, including chao1, observed operational taxonomic units (OTUs) and phylogenetic diversity whole tree (PD whole tree), respectively. Microbiota diversities were obviously reduced in *Tcf7*^−/−^ mice at 5 and 22 weeks old. However, diversities were not statistically significant in 10 weeks old *Tcf7*^−/−^ mice, though the similar tendency was also exhibited (Fig. S3A). Regarding the relative abundance of OTUs at the phylum level, higher *Bacteroidetes* and lower *Firmicutes* were exhibited in the microbial community of *Tcf7*^−/−^ mice than their littermate controls (Fig. 1F). We further applied linear discriminant analysis (LDA) and linear discriminant analysis of effect size (LEfSe) to the microbiome data and found several differentially abundant clades in control and *Tcf7*^−/−^ mice (Fig. S3B and S3C). For example, *Tcf7*^−/−^ mice had increased levels of inflammation promoting bacteria including *Prevotellaceae, Akkermansia* and *Enterococcus*, and decreased levels of healthy bacteria such as *Lactobacillus* and *Bifidobacterium* (Fig. S3B and S3C). Next, the abundances of representative bacteria belong to several taxonomic groups were shown at phylum, class, family and genus levels, respectively. In addition, an increase of *Bacteroides* (dominant in *Bacteroidetes*) at the genus level in 10 weeks old group and a decrease in *Bacilli* (dominant in *Firmicutes*) at the class level in 10 and 22 weeks were found in *Tcf7*^*−/−*^ mice (Fig. 1G). To detect the synergy effects of taxa, the abundance-based correlation networks of enriched OTUs were analyzed between control and *Tcf7*^−/−^ mice using the data from 5, 10 and 22 weeks old groups, respectively. As expected, a number of enriched OTUs in control samples are negatively correlated with those in *Tcf7*^−/−^ samples, reflecting that the entire alteration of the flora occurred in the intestinal contents of the *Tcf7*^−/−^ mice. Meanwhile, the enriched OTUs inside of the control or TCF-1 deficient subjects have shown positive correlations (Fig. S3D). Our results also demonstrated that the alteration of complexity between control and *Tcf7*^−/−^ mice is associated with age.

To assess the functional effects based on the diversity alteration of intestine microbial community, KEGG pathway analysis was performed using the 16S rRNA sequencing data. There were more different pathways in the 22 weeks old group than in 5 or 10 weeks old groups, in particular, those immune and infection related pathways. For example, the pathway of infectious diseases (PATH: ko09171) was significantly different between *Tcf7*^−/−^ mice and controls at 10 and 22 weeks old, but not at 5 weeks old. Our results indicated that these significant differences were exhibited gradually in various pathways along with age (Fig. S3E). One possible reason is the long-term interactions between host and microbiota in different genetic environment, establishing the homeostasis of different gut microbiota composition. On the other side, elderly mice have decreased intestinal function compare to the younger, including digestion, nutrient absorption and immune activity (Odamaki et al, 2016). These integrated factors accumulate more differences between elderly TCF-1 deficient and control groups (Donaldson et al, 2016). These findings offer an insight to clarify the gut microbiota composition in genetically defective host at different age period.

To explore the relevance between the dysbiosis and intestinal inflammation due to TCF-1 deficiency, we set up induced colitis models with the antibiotic-pretreated wild type mice receiving fecal from different donors as shown in the flowchart (Fig. 2A). As reference, we first induced the colitis models with *Tcf7*^−/−^ mice or co-housed controls by dextran sulfate sodium (DSS). *Tcf7*^−/−^ mice exhibited more body weight loss and more severe gut inflammation than their co-housed controls in response to DSS challenge (Fig. 2B and 2C, left panel). In consistent with the previous study (Xing et al, 2019), these results indicated that TCF-1 deficiency leads to enhanced colitis. However, these severe colitis symptoms are not only originated from dysbiosis since the intestinal inflammation is associated with multiple complicated factors. Subsequently, the induced colitis models were established with antibiotic-pretreated wild type mice received fecal microbiota from 22 weeks old *Tcf7*^−/−^ mice or co-housed controls. We found that *Tcf7*^−/−^-mouse-derived fecal microbiota recipients lost more body weight and had more severe symptoms than their co-housed controls in response to DSS challenge (Fig. 2B and 2C, right panel). The colons of *Tcf7*^−/−^-mouse-derived fecal microbiota recipients were shorter than control mice (Fig. 2D and 2E). Accordingly, the recipient mice received *Tcf7*^−/−^-mouse-derived fecal microbiota displayed more crypt architecture loss and destructive tissue damage (Fig. 2F and 2G). These data demonstrated more severe colonic inflammation was developed in *Tcf7*^−/−^ mouse derived fecal microbiota recipients than their controls, and the dysbiosis of gut microbiota due to TCF-1 deficiency is an indispensable factor to enhance intestinal inflammation upon DSS challenge.

**Figure 2:**
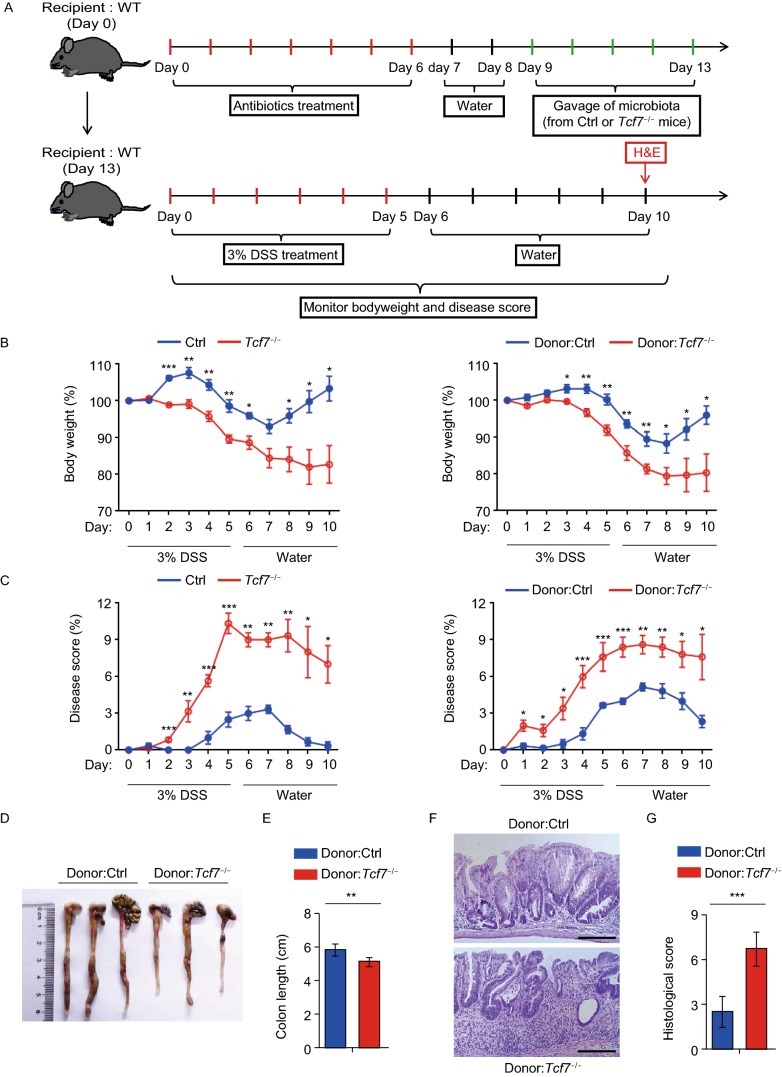
**Recipients with*Tcf7***^**−/−**^**mice microbiota develop more severe DSS-induced colitis**. (A) Flowchart for generating fecal-microbiota recipients followed by 3% DSS treatment. (B, left) Body weight of *Tcf7*^−/−^ mice and controls was summarized after 3% DSS treatment (*n* ≥ 3). (C, left) Disease scores reflecting symptom of DSS-induced colitis with *Tcf7*^−/−^ mice and controls (*n* ≥ 6). Body weight (B, right) and disease scores (C, right) of recipients with *Tcf7*^−/−^ or control mice microbiota were shown at indicated time points (*n* ≥ 5 for each group). (D) Representative colon length of recipient mice and statistical data (E) were shown at day 10 after 3% DSS challenge (*n* ≥ 5 for each group). (F) Representative H&E staining of colon sections and pathological scores from recipients with *Tcf7*^−/−^ or control mice microbiota at day 10 post 3% DSS challenge were summarized in (G). Scale bars, 200 μm. Data are mean ± SD and representative of at least two independent experiments. *P < 0.05, **P < 0.01, ***P < 0.001, (Student’s *t*-test)

In summary, *Tcf7*^−/−^ mice at different age stages were used to evaluate the role of TCF-1 in intestinal microbiota and 16S rRNA sequencing data reflected that the composition of gut bacterial community was disorder in *Tcf7*^−/−^ mice. Compared with their co-housed littermate controls, TCF-1-deficient mice exhibited no visible PPs, smaller MLNs and diminished GCBs correlated with defects of germinal center responses, resulting in a defective IgA production and a decrease of IgA-binding bacteria. Accordingly, the dysbiosis of gut bacteria community in *Tcf7*^−/−^ mice is associated with age and contributes to enhancing colonic inflammation. Thus, our results uncovered the previously unknown role of TCF-1 in maintaining homeostasis of intestinal microbiota and demonstrated TCF-1 deficiency as a risk factor for gut inflammation with potential implication on clinical trials.

## FOOTNOTES

We thank Drs Shiqi Luo and Hao Zheng at Beijing Advanced Innovation Center for Food Nutrition and Human Health, China Agricultural University for their advice on analyzing high throughput data. We thank Drs Xuekun Guo and Xiaoyu Hu at Institute for Immunology and School of Medicine, Tsinghua University for their constructive suggestion on DSS induction experiments. We thank Dr. Jincun Zhao at Guangzhou Medical University for providing *act*A^−^LM-Ova. We thank Dr. Bing Zhang at the Laboratory Animal Center in China Agricultural University for his assistant of animal care.

This work is supported by National Key R & D Program of China (2017YFA0104401) to S.Y. and National Natural Science Foundation of China (Grant Nos. 31571522, 31970831, 31630038 and 31422037) to S.Y. and the Project for Extramural Scientists of State Key Laboratory of Agrobiotechnology from China Agricultural University, China (2019SKLAB6-6).

S.Y. and F.C. designed and supervised the experiments. G.Y. and M.Y. performed the major experiments and analyzed overall experimental data; F.W., T.X., and M.Y. analyzed and presented the high throughput data. C.S., R.L., M.D., Y.L., Z.Q., Z.W., J.L., Y.Y., J.C., Z.S., S.H., W.G., T.Z., Z.Y., Q.Z. and Y.Z. assisted the overall experiments and interpreted data. G.Y., F.W., T.X., and S.Y. wrote the manuscript with the revision of all authors.

Guotao Yu, Fang Wang, Menghao You, Tiansong Xu, Chunlei Shao, Yuning Liu, Ruiqi Liu, Min Deng, Zhihong Qi, Zhao Wang, Jingjing Liu, Yingpeng Yao, Jingjing Chen, Zhen Sun, Shanshan Hao, Wenhui Guo, Tianyan Zhao, Zhengquan Yu, Qian Zhang, Yaofeng Zhao, Feng Chen, Shuyang Yu declare that they have no conflict of interest. All institutional and national guidelines for the care and use of laboratory animals were followed.

## Electronic supplementary material

Below is the link to the electronic supplementary material.

Supplementary material 1 (PDF 5217 kb)
