# Supplementary material for: TCF-1 deficiency influences the composition of intestinal microbiota and enhances susceptibility to colonic inflammation
Source: Protein Cell. 2020 Jan 22;11(5):380–6. doi: 10.1007/s13238-020-00689-8 (PMC7196568; doi:10.1007/s13238-020-00689-8)
Supplement: Supplementary file 1 — Supplementary material 1 (PDF 5217 kb) [file 13238_2020_689_MOESM1_ESM.pdf]

Materials and Methods

Mice

The animal experiments were approved by the Institutional Animal Care and Use Committee at China Agricultural University. *Tcf7*$^{-/-}$ mice were originally obtained from Dr. Hai-Hui Xue and described as previously (Verbeek et al, 1995). All mice used in this study were on a fully C57BL/6J background at 5, 10 and 22 weeks old. All experiments were performed using littermate animals from heterozygous breeding pairs. To exclude the possibility that certain environmental factors may influence the microbiota, mice were bred and group housed in specific pathogen-free conditions under controlled temperature (22±1 °C) and exposed a constant 12 hour light-dark cycle.

Cell isolation, staining and flow cytometry

Single-cell suspensions of peripheral blood cells (PBCs), spleens (SPs) or mesenteric lymph nodes (MLNs) were used for flow cytometry analysis or cell sorting. Cell isolation, staining and analysis by flow cytometry were performed as previously described (Cui et al, 2017). Simply, cells were stained with fluorochrome-conjugated antibodies in PBS containing 2% FBS. The following fluorescence-labeled monoclonal antibodies were used: anti-CD4 (RM4-5), anti-CD138 (281-2) (from BD Horizon); anti-B220 (RA3-6B2), anti-CD19 (1D3), anti-CD44 (IM7), anti-CD62L (MEL-14), anti-GL7 (GL7), anti-PD-1 (J43), (eBioscience); anti-Fas (Jo2, BD Biosciences), anti-SLAM (TC15-12F12.2, Biolegend) and anti-PNA (Cat # FL-1071, from Vector Laboratories). CXCR5 staining was performed with a three-steps staining protocol. Briefly, single-cell suspensions were first stained with purified anti-CXCR5 (2G8; BD Biosciences) for 1 h, followed by biotin-conjugated goat anti-rat IgG (112-066-143; Jackson Immunoresearch) for 30 min, and then by APC-Cy7-labeled streptavidin (eBioscience) at 4°C for 30 min in PBS supplemented with 2% normal mouse serum (015-000-120; Jackson Immunoresearch), 2% FBS, and 0.5% BSA. Flow cytometric assay was performed with a FACSVerse (BD Biosciences) cytometer and the data were analyzed with FlowJo software (Treestar). Cell sorting was performed with a FACSAria (BD Biosciences), following the procedures as previously described (Liu et al, 2019).

Analysis of IgA-bound bacteria

Fecal pellets were collected and homogenized in 1000 µl of sterile 1× PBS. Samples were spun at 400 × g to remove course materials from the fecal suspension. Supernatants (containing bacteria) were collected and placed in a new 1.5 ml tube. Samples were spun at 8000 × g for 5 minutes to pellet bacteria.

Bacterial suspensions were then blocked on ice for 15 minutes in 100 µl sterile 1× PBS containing 1% BSA. Bacterial pellets were next stained for 20 minutes at 4 °C in the dark in 500 µl of sterile 1× PBS containing a 1:300 dilution of a PE-conjugated rat anti-mouse IgA antibody (eBioscience, Cat # 12-5994-81). Pellets were washed twice as above in sterile 1× PBS containing 1% BSA. After washing, samples were analyzed on a FACSVerse (BD Biosciences).

Enzyme linked immunosorbent assay (ELISA)

Fecal samples were collected from mice at 5, 10 and 22 weeks old. 0.1 g of feces were soaked in 1 ml PBS at 4 °C for 2 h and spun at 400 × g for 5 minutes to remove large particles from bacteria. Supernatants were then transferred into a new tube and spun at 8000 × g for 5 minutes to pellet bacteria. This step was repeated until samples were clear of bacterial pellets. Total IgA levels in feces were measured as previously described (Yao et al, 2018). In brief, each fecal sample was diluted at the appropriate concentration (IgA, 1:80; IgG, 1:10). Then all samples were coated with the goat anti-mouse IgA (A90-103A-30, 1:400 dilution) or goat anti-mouse IgG (A90-131A-17, 1:400 dilution) followed by HRP-conjugated goat anti-mouse IgA (A90-103P-34, 1:30000 dilution) or HRP-conjugated goat anti-mouse IgG (A90-131P-39, 1:100000 dilution). For quantification of OVA specific IgA, instead of coating plates with capture antibody specific for IgA, plates were coated overnight at 4°C with 0.5 mg/ml ovalbumin in 1 × PBS. The absorbance at 450 nm was measured with a microplate reader (TECAN).

16S rRNA gene amplification and sequencing

Fecal samples from *Tcf7*$^{-/-}$ or control mice were collected in PBS and stored at -80 °C until processing. Bacterial DNA was extracted using PowerSoil DNA Isolation Kit (MoBio Laboratories, Carlsbad, CA) following the manufacturer's instruction. The purity and quality of the genomic DNA were checked on 0.8% agarose gels. Fecal samples of mice were collected and processed as above for sequencing. To obtain high-quality sequencing data, we amplified the hypervariable regions 3 and 4 of bacterial 16S rRNA genes with the amplification primers 338F (ACTCCTACGGGAGGCAGCAG) and 806R (GGACTACHVGGGTWTCTAAT). The PCR amplicons were sequenced on an Illumina MiSeq with paired-end 300-cycle sequencing at Allwegene Company (Beijing, China). The data are available in the Sequence Read Archive under accession number SRP211961.

16S rRNA data processing and microbiome analysis

Raw data of 16S rRNA sequences were de-multiplexed with a custom script. Reads with an average

quality value of <20 were filtered off and sequences which had any 'N' nucleotides were discarded. Next, cleaned paired-end reads were merged using the FLASH based on overlapping sequences after trimming off both primer sequences. Quality-filtered reads were processed and analyzed with Quantitative Insights Into Microbial Ecology 1.9.1 (QIIME 1.9.1) and R software. The sequences were assigned to operational taxonomic units (OTUs) at a level of 97% similarity using ribosomal database project (RDP) and made taxonomic calls against the Greengenes (v13_8) reference set and taxonomy. Representative sequences were aligned using UCLUST and chimera sequences were screened using ChimeraSlayer and then removed with the default parameter setting of QIIME. Taxonomy was assigned using RDP classifier with a confidence threshold 0.5. OTU tables were used to estimate alpha and beta diversity using QIIME1 scripts. Alpha diversity of observed species was measured by Chao1 index, observed OTUs and PD whole tree index. Fixed numbers of sequences were randomly selected from each dataset and allow the microbial diversity to be evaluated. To compare the microbial communities based on their composition, we employed the QIIME default UniFrac distance metric. The data were visualized with principal coordinate analysis (PCoA). Correlation networks were visualized using Cytoscape (ver. 3.7.2). Spearman's correlation coefficient and the statistical significance were calculated by using R package. Edges were set between pairs of OTUs (coefficient $\leq$ -0.8 or $\geq$0.8; $P < 0.05$).

Bacteria functional predictions were achieved from the Kyoto Encyclopedia of Gene and Genomes (KEGG) catalog using the Tax4Fun program and custom scripts. The R project for statistical computing (https://www.r-project.org) was used to perform functional (KEGG level 2) comparison between control and *Tcf7*$^{-/-}$ mice samples. Significant functional differences were determined by wilcoxon test (*$P < 0.05$; ** $P < 0.01$; *** $P < 0.001$). To assess possible OTUs associated with each group (control and *Tcf7*$^{-/-}$ mice at 5, 10 and 22 weeks old), linear discriminant analysis effect size (LEfSe) was performed via the Galaxy web application. Bacterial abundance profiles were calculated at taxonomic levels from phylum to genus in percent abundance and alpha values $\geq$ 0.05 (Kruskal-Wallis test) and a logarithmic linear discriminant analysis (LDA) score $\geq$ 2.0 were used as thresholds between control and *Tcf7*$^{-/-}$ mice at 5, 10 and 22 weeks old, respectively.

***act*A^{-}LM-Ova infection**

Attenuated LM-Ova strain (referred to as *act*A^{-}LM-Ova) was a gift from Dr. Jincun Zhao (Guangzhou Medical University) and was created by introducing an in-frame deletion in the *act*A gene.

Briefly, *act*A$^-$LM-Ova bacteria were cultured in Tryptic Soy Broth with Yeast Extract (TSB-YE) with streptomycin (Solarbio) at a concentrate of 50 μg/ml. Cultures were grown in a 37°C orbital shaking incubator until the late exponential phase of growth, when the optical density (at 600 nm) of the culture was 0.6. *Tcf7*$^{-/-}$ mice and littermate controls were *i.p.* infected with 8×10^7 CFU *act*A$^-$LM-Ova, and Tfh cells differentiation and GC response were analyzed on day 7 after infection.

Immunofluorescence staining.

Spleen and MLN tissues were placed on bibulous paper to remove excess liquid and submerged in mold with OCT compound (4583; Sakura), and then were quickly frozen in liquid nitrogen and cut into 10-μm-thick sections. Frozen tissue sections were then fixed in pre-chilled acetone for 30 min at −20°C, followed by blocking with 5% BSA and Fc-blocker (2.4G2; BD Biosciences) in PBS for 30 min. The sections were then stained with biotin-PNA (Cat # FL-1071; Vector laboratories), followed by staining with PE-conjugated B220 (Cat# RA3-6B2; eBioscience) and FITC-conjugated streptavidin (Cat # S32354; Invitrogen). Among every step, the slides were washed at least three times with PBS. After Immunofluorescence staining, one drop of Antifade Mounting Medium was added to the slide and mounted by coverslips (Cat # P012; Beyotime). The signals were examined by a Zeiss LSM 800 confocal fluorescence microscope and the images were processed with LSM Image Examiner software (Zeiss).

Fecal microbiota transplantation and DSS-Induced colitis

For fecal-microbiota transplantation experiments, 8 to 10-week-old recipient mice (wild type mice) were treated with purified drinking water containing antibiotics cocktails of 1 mg/ml of ampicillin (Solarbio), 0.5 mg/ml of vancomycin (Solarbio), 1 mg/ml of neomycin sulphate (Solarbio) and 1 mg/ml of metronidazole (Solarbio) for one week. Antibiotic treatment was then terminated and switched to autoclaved drinking water for 2 days. Then recipient mice were orally gavaged daily for 5 days with 100 μl of microbiota stock derived from 20 to 22-week-old *Tcf7*$^{-/-}$ mice or their co-housed control mice. Microbiota stocks prepared from fresh feces (1 g) that were resuspended in 1 ml sterile PBS and centrifuged at 400 × g for 5 minutes to remove large particles. Five days post microbiota transplantation, recipient mice were given sterile and distilled water containing 3% DSS (MP Biomedicals) for 5 consecutive days. After DSS treatment, mice were fed with regular water. Body weight, stool softness, and blood in the rectum or fur were recorded daily and the disease activity index (DAI) was monitored. Briefly, disease score was defined as follows: (1) weight loss (no change = 0; < 5% = 1; 6–10%= 2; 11–20% = 3; >

20% = 4); (2) feces (normal = 0; pasty, semiformed = 2; liquid, sticky, or unable to defecate after 5 min = 4); (3) blood (no blood = 0; visible blood in rectum = 1; visible blood on fur = 2); and (4) general appearance (normal = 0; piloerection = 1; lethargy and piloerection = 2; motionless = 4).

Histological scoring

After 5 or 10 days of DSS administration, mice were sacrificed and the entire colon was excised and measured. Tissues were fixed in 4% paraformaldehyde, paraffin embedded, and sectioned (4 μm). Sections were stained with hematoxylin and eosin for immunofluorescence. Histological scores were assigned by experimenters “blinded” to sample identity. Epithelial damage of colon was assessed scores as follows: 0 = normal; 1 = hyperproliferation, irregular crypts, and goblet cell loss; 2 = mild to moderate crypt loss (10–50%); 3 = severe crypt loss (50–90%); 4 = complete crypt loss, surface epithelium intact; 5 = small- to medium-sized ulcer (<10 crypt widths); 6 = large ulcer (≥10 crypt widths). Infiltration with inflammatory cells was assigned scores separately for mucosa (0 = normal, 1 = mild, 2 = modest, 3 = severe) and submucosa (0 = normal, 1 = mild to modest, 2 = severe). Scores for epithelial damage and inflammatory cell infiltration were accumulated and the total scores from 0 to 12 were summarized for indicating the degree of pathological damage.

Statistical analysis

Statistical analysis was performed using unpaired two-tailed Student’s t-test with Prism 6 (GraphPad Software) unless stated otherwise. T-test with a 95% confidence interval was used to calculate P values. P values ≤ 0.05 were considered significant (*$P < 0.05$; ** $P < 0.01$; *** $P < 0.001$); P values > 0.05 were considered non-significant (ns). Results are presented as mean ± SD.

References

Cui Z, Niu S, Liu J, Xu L, Dai Y, Li N, Kang Y, Zhang L, Zhou L, Yu S (2017) Over-expression of CD163, CD169, and CD151 is not sufficient to improve the susceptibility to porcine reproductive and respiratory syndrome virus infection in transgenic mice. Science Bulletin 62:1634-1636

Liu J, Cui Z, Wang F, Yao Y, Yu G, Liu J, Cao D, Niu S, You M, Sun Z, Lian D, Zhao T, Kang Y, Zhao Y, Xue HH, Yu S (2019) Lrp5 and Lrp6 are required for maintaining self-renewal and differentiation of hematopoietic stem cells. FASEB J 33:5615-5625

Verbeek S, Izon D, Hofhuis F, Robanus-Maandag E, te Riele H, van de Wetering M, Oosterwegel M, Wilson A, MacDonald HR, Clevers H (1995) An HMG-box-containing T-cell factor required for thymocyte differentiation. Nature 374:70-74

Yao Y, Guo W, Chen J, Guo P, Yu G, Liu J, Wang F, Liu J, You M, Zhao T, Kang Y, Ma X, Yu S (2018) Long noncoding RNA Malat1 is not essential for T cell development and response to LCMV infection. RNA Biol

Yu et al. Supplementary figure1

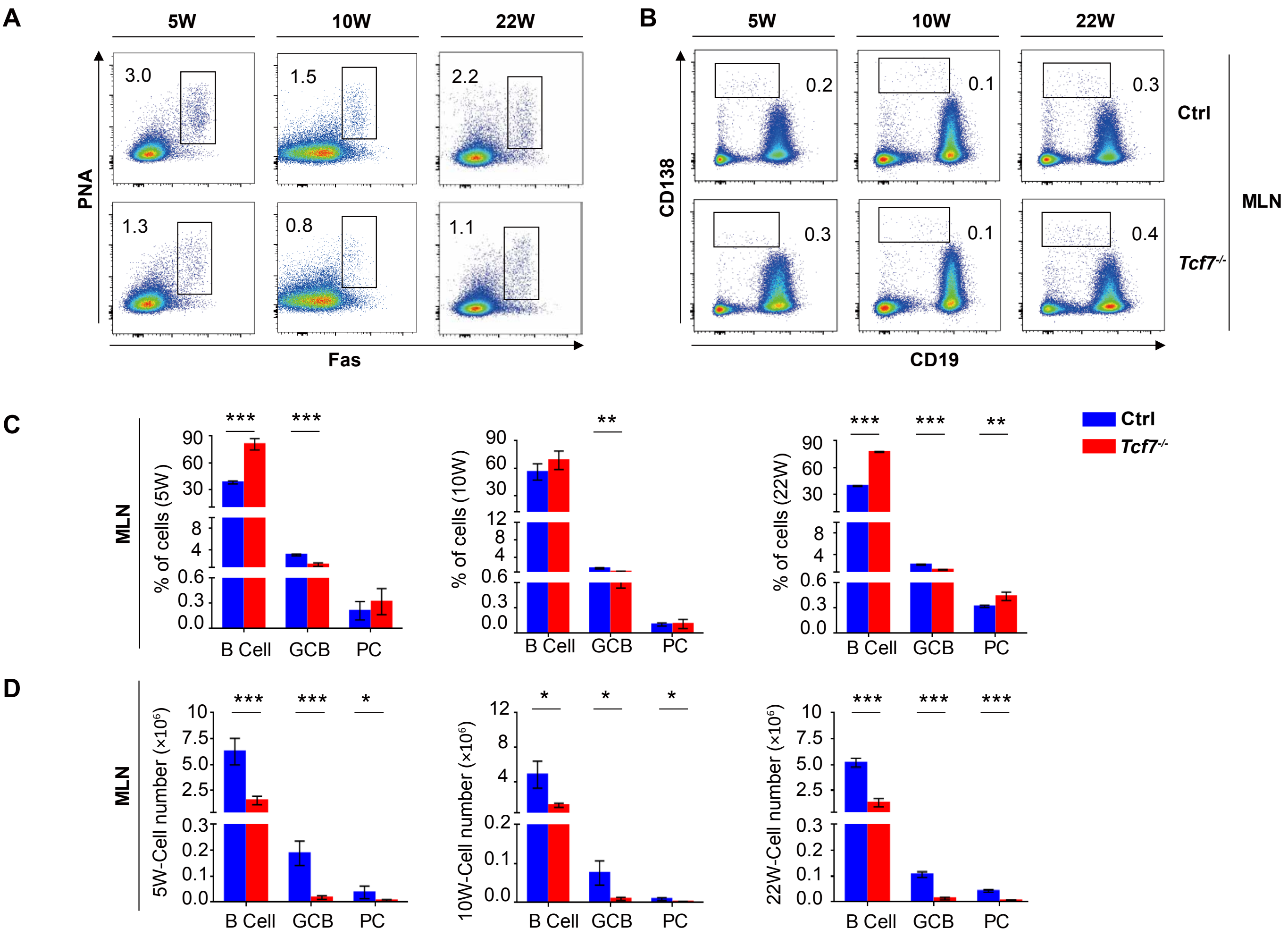

Fig. S1: Ablation of TCF-1 leads to reduced GCBs and PCs in MLNs.

Flow cytometry analysis of $B220^+CD19^+PNA^+Fas^+$ GCBs (A) and $CD138^+CD19^{lo}$ plasma cells (PCs) (B). Frequencies of B cells, GCBs and PCs in MLNs were shown statistically in (C) ($n \geq 4$ for each group). (D) The numbers of B cells, GCBs and PCs in MLNs were shown statistically ($n \geq 4$ for each group). Data are mean ± SD and representative of two independent experiments. *$P < 0.05$, **$P < 0.01$, ***$P < 0.001$ (Student's *t*-test).

Yu et al. Supplementary figure2

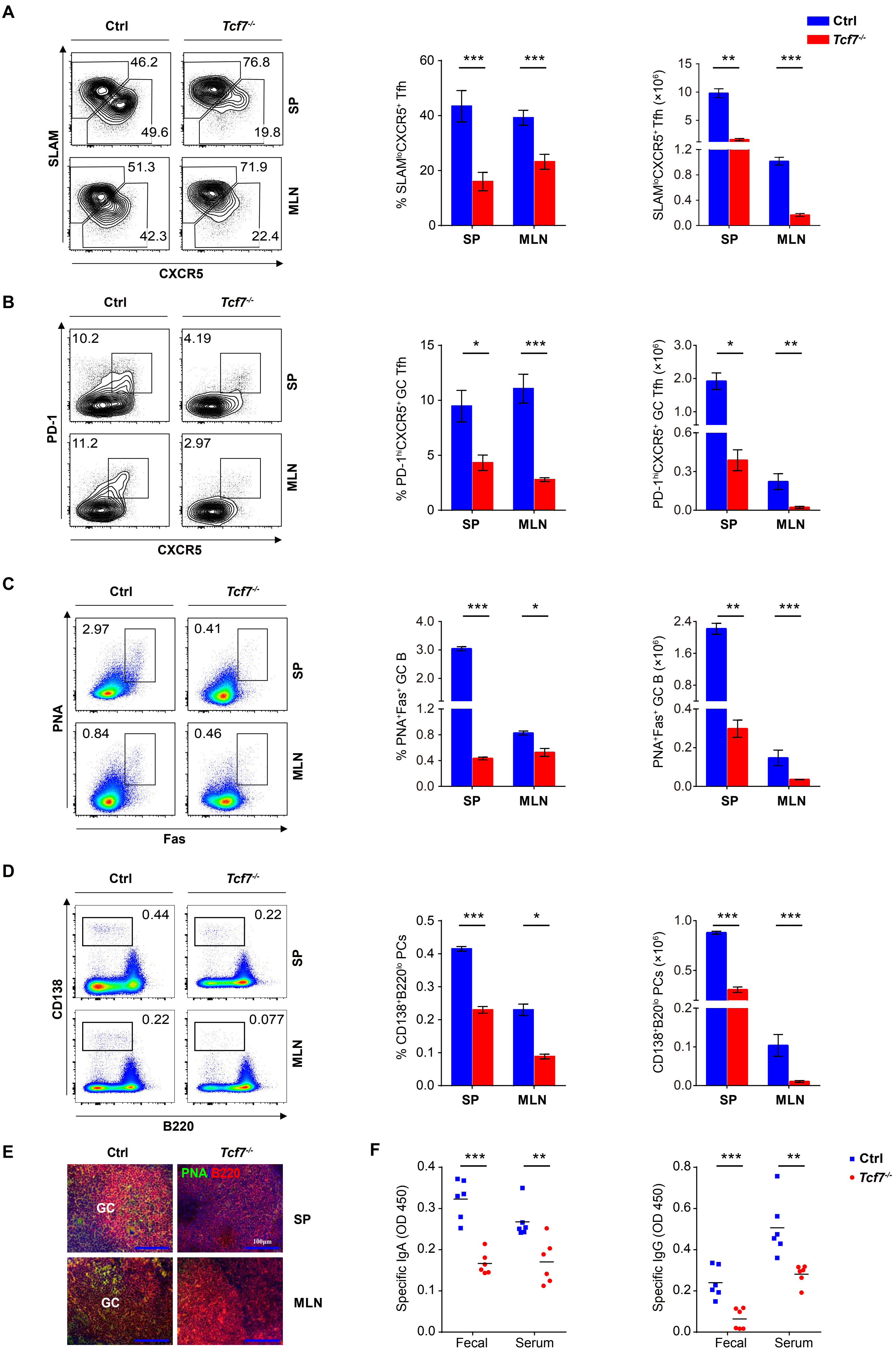

Fig. S2: TCF-1 deficiency results in defects of Tfh cells differentiation and GC response upon *act*A$^-$LM-Ova challenge

(A) Representative flow cytometry contour plots of SLAM^{lo}CXCR5$^+$ Tfh cells gated on CD4$^+$CD44$^+$ CD62L$^-$ T cells in SPs and MLNs from indicated genotype on the day 7 after infection. The corresponding frequencies and numbers were statistically shown (n ≥ 4 for each group). (B) Representative flow cytometry contour plots of PD-1^{hi}CXCR5$^+$ GC Tfh cells. The corresponding frequencies and numbers were statistically shown (n ≥ 4 for each group). (C) Flow cytometry analysis of GCBs (PNA$^+$Fas$^+$CD19$^+$B220$^+$) in SPs and MLNs from indicated genotype. The corresponding frequencies and numbers were statistically shown (n ≥ 4 for each group). (D) Flow cytometry analysis of plasma cells (CD138$^+$B220^{lo}) in SPs and MLNs from indicated genotype. The corresponding frequencies and numbers were statistically shown (n ≥ 4 for each group). (E) Representative immunohistochemistry of B220 and PNA in spleens and MLNs from *act*A$^-$LM-Ova infected *Tcf7*$^{-/-}$ mice and their littermate controls. (F) Ova-specific IgA and IgG concentration in cecal content at day 7 after *act*A$^-$LM-Ova infection. Each symbol represents an individual sample (n = 6 for each group). Data are mean ± SD and representative of two independent experiments. *P < 0.05, **P < 0.01, ***P < 0.001, (Student's *t*-test).

Yu et al. Supplementary figure3

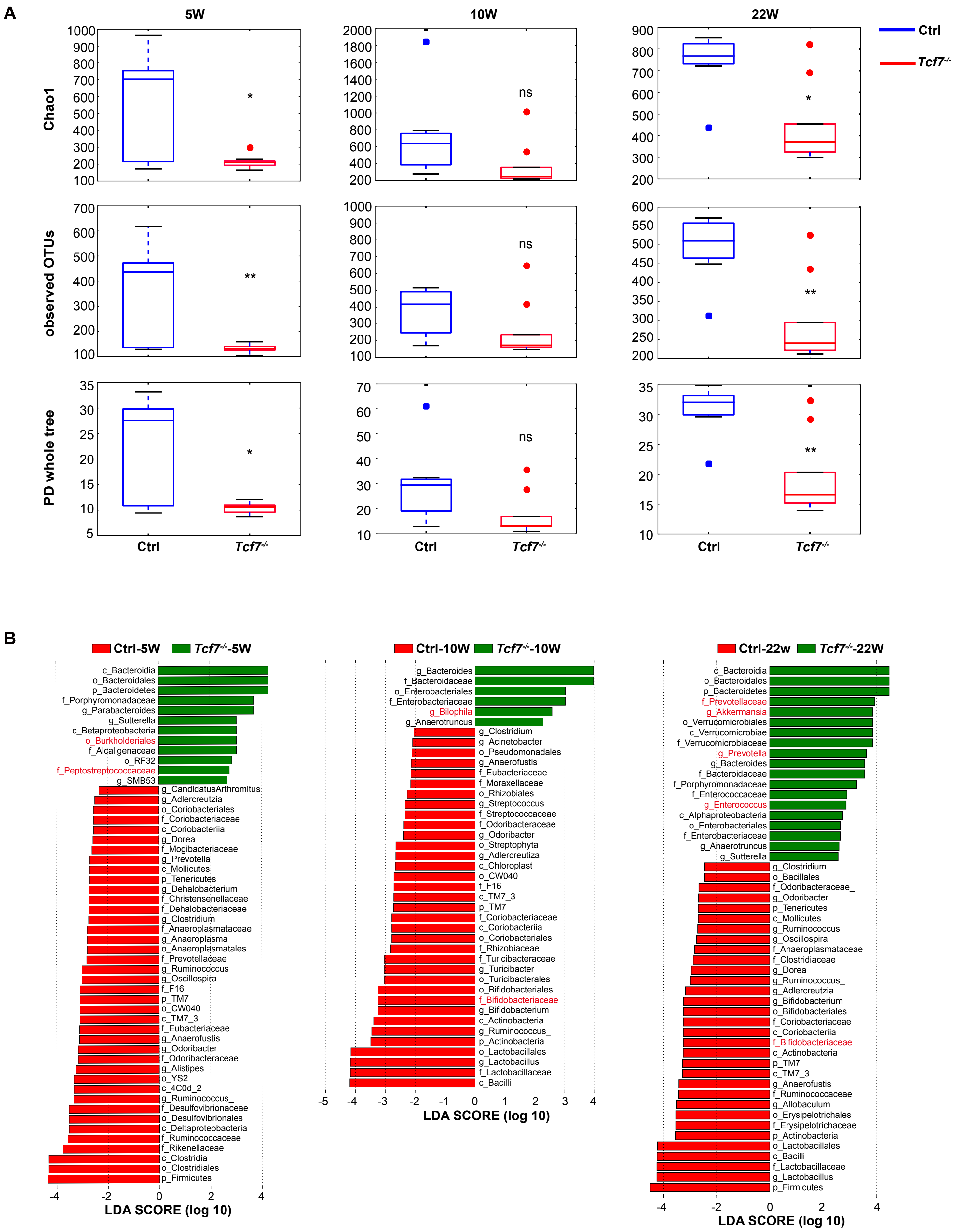

Yu et al. Supplementary figure3

C

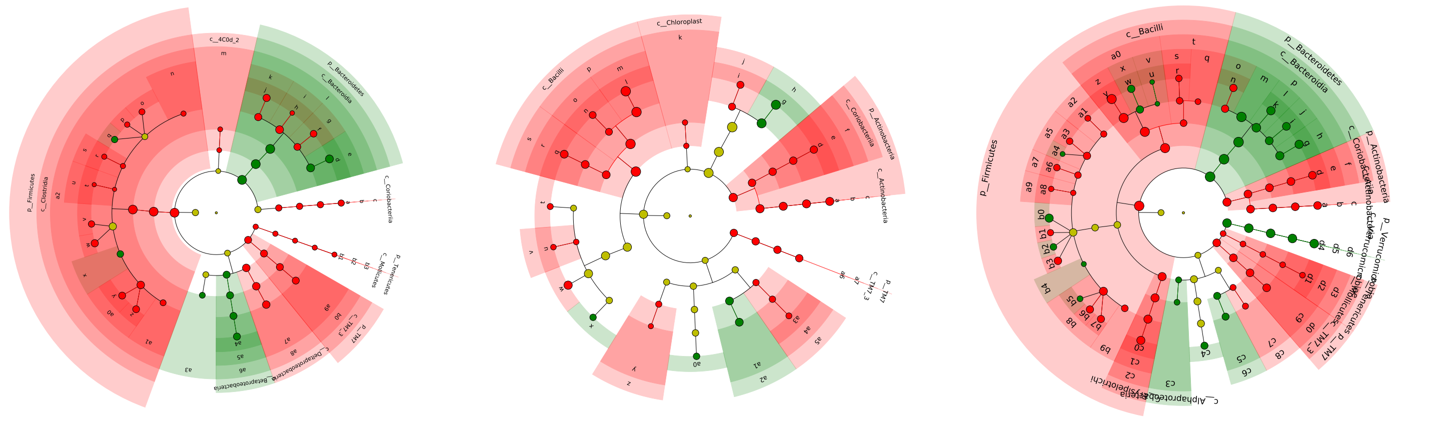

Ctrl-5w *Tcf7*$^{-/-}$-5w

a: g__Adlercreutzia
b: f__Coriobacteriaceae
c: o__Coriobacteriales
d: g__Parabacteroides
e: f__Porphyromonadaceae
f: g__Prevotella
g: f__Prevotellaceae
h: g__Alistipes
i: f__Rikenellaceae
j: g__Odoribacter
k: f__Odoribacteraceae_
l: o__Bacteroidales
m: o__YS2
n: f__Christensenellaceae
o: g__CandidatusArthromitus
p: g__Clostridium
q: g__SMB53
r: g__Dehalobacterium
s: f__Dehalobacteriaceae
t: g__Anaerofustis
u: f__Eubacteriaceae
v: g__Dorea
w: g__Ruminococcus_
x: f__Peptostreptococcaceae
y: g__Oscillospira
z: g__Ruminococcus
a0: f__Ruminococcaceae
a1: f__Mogibacteriaceae_
a2: o__Clostridiales
a3: o__RF32
a4: g__Sutterella
a5: f__Alcaligenaceae
a6: o__Burkholderiales
a7: f__Desulfovibrionaceae
a8: o__Desulfovibrionales
a9: f__F16
b0: o__CW040
b1: g__Anaeroplasma
b2: f__Anaeroplasmataceae
b3: o__Anaeroplasmatales

Ctrl-10w *Tcf7*$^{-/-}$-10w

a: g__Bifidobacterium
b: f__Bifidobacteriaceae
c: o__Bifidobacteriales
d: g__Adlercreutzia
e: f__Coriobacteriaceae
f: o__Coriobacteriales
g: g__Bacteroides
h: f__Bacteroidaceae
i: g__Odoribacter
j: f__Odoribacteraceae_
k: o__Streptophyta
l: g__Lactobacillus
m: f__Lactobacillaceae
n: g__Streptococcus
o: f__Streptococcaceae
p: o__Lactobacillales
q: g__Turicibacter
r: f__Turicibacteraceae
s: o__Turicibacterales
t: g__Clostridium
u: g__Anaerofustis
v: f__Eubacteriaceae
w: g__Ruminococcus_
x: g__Anaerotruncus
y: f__Rhizobiaceae
z: o__Rhizobiales
a0: g__Bilophila
a1: f__Enterobacteriaceae
a2: o__Enterobacteriales
a3: g__Acinetobacter
a4: f__Moraxellaceae
a5: o__Pseudomonadales
a6: f__F16
a7: o__CW040

Ctrl-22w *Tcf7*$^{-/-}$-22w

a: g__Bifidobacterium
b: f__Bifidobacteriaceae
c: o__Bifidobacteriales
d: g__Adlercreutzia
e: f__Coriobacteriaceae
f: o__Coriobacteriales
g: g__Bacteroides
h: f__Bacteroidaceae
i: g__Parabacteroides
j: f__Porphyromonadaceae
k: g__Prevotella
l: f__Prevotellaceae
m: f__S24_7
n: g__Odoribacter
o: f__Odoribacteraceae_
p: o__Bacteroidales
q: f__Planococcaceae
r: g__Jeotgalicoccus
s: f__Staphylococcaceae
t: o__Bacillales
b4: f__Peptostreptococcaceae
b5: g__Anaerotruncus
b6: g__Oscillospira
b7: g__Ruminococcus
b8: f__Ruminococcaceae
b9: f__Mogibacteriaceae_
c0: g__Allobaculum
c1: f__Erysipelotrichaceae
c2: o__Erysipelotrichales
c3: o__RF32
c4: g__Sutterella
c5: f__Enterobacteriaceae
u: g__Granulicatella
v: f__Carnobacteriaceae
w: g__Enterococcus
x: f__Enterococcaceae
y: g__Lactobacillus
z: f__Lactobacillaceae
a0: o__Lactobacillales
a1: g__Christensenella
a2: f__Christensenellaceae
a3: g__Clostridium
a4: g__SMB53
a5: f__Clostridiaceae
a6: g__Dehalobacterium
a7: f__Dehalobacteriaceae
a8: g__Anaerofustis
a9: f__Eubacteriaceae
b0: g__Blautia
b1: g__Dorea
b2: g__Roseburia
b3: g__Ruminococcus_
c6: o__Enterobacteriales
c7: f__Moraxellaceae
c8: o__Pseudomonadales
c9: f__F16
d0: o__CW040
d1: g__Anaeroplasma
d2: f__Anaeroplasmataceae
d3: o__Anaeroplasmatales
d4: g__Akkermansia
d5: f__Verrucomicrobiaceae
d6: o__Verrucomicrobiales

Yu et al. Supplementary figure3

D

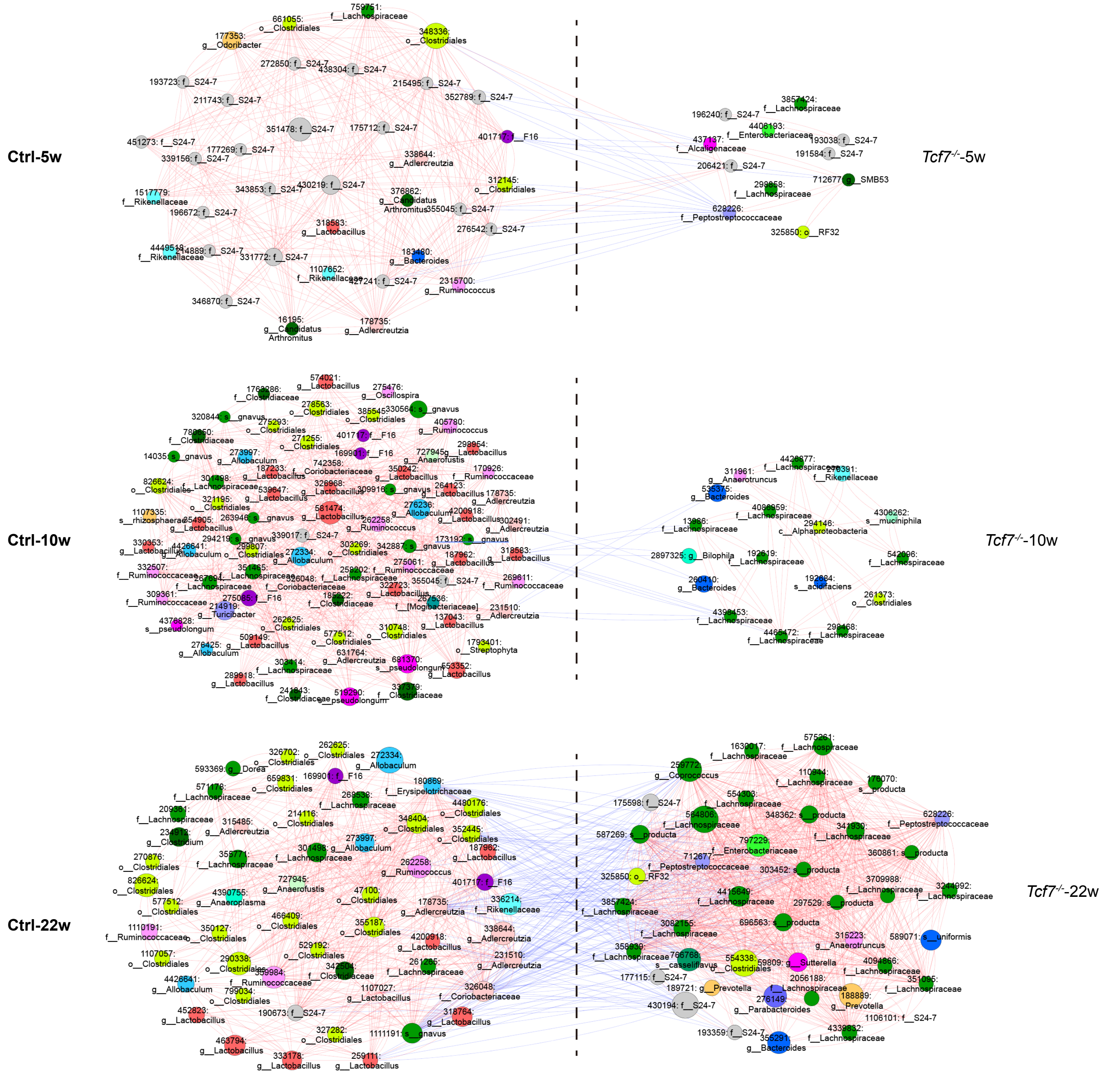

E

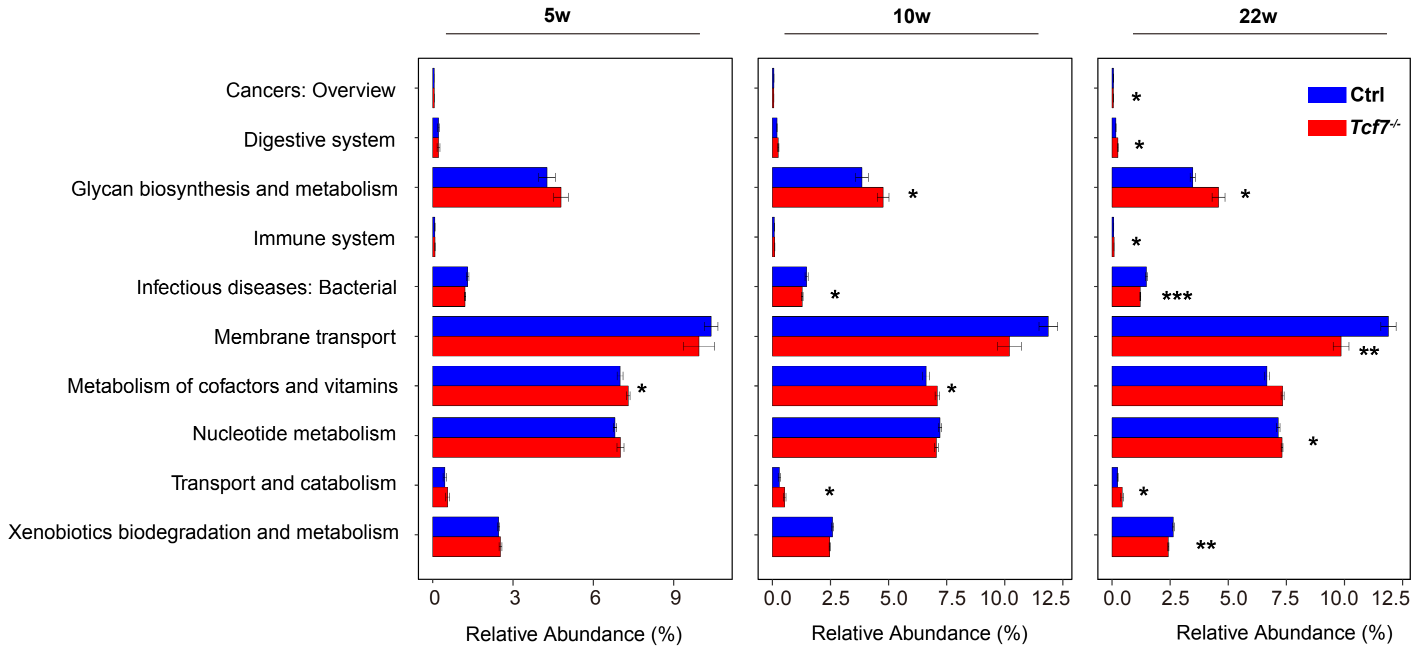

Fig. S3: Distinct fecal microbiota composition in *Tcf7*$^{-/-}$ mice.

(A) Alpha diversity analysis. Chao1, Observed OTUs and PD whole tree in the intestinal bacterial communities (n = 8). (B) Histogram showing the linear discriminant analysis (LDA) scores distribution of the differentially abundant taxa between *Tcf7*$^{-/-}$ mice and controls at 5, 10, and 22 weeks old (taxa with LDA score ≥ 2.0 and significance of α < 0.05 determined by Wilcoxon test) (n = 8 for each group). (C) Linear discriminant analysis of effect size (LEfSe) plots highlight significantly different abundant microbial clades enriched in microbiota from feces of *Tcf7*$^{-/-}$ mice or control mice (n = 8 for each group). (D) Correlation networks of differentially abundant OTUs from Ctrl and *Tcf7*$^{-/-}$ mice at 5, 10 and 22 weeks old. Pearson's correlation was used for calculation (coefficients ≤ -0.8 or ≥0.8; P < 0.05,). Each node in the network represents an individual OTU. The node size is proportional to the mean relative abundance and the color indicates the different genus. (E) KEGG pathways reflected the differences in potential functions between *Tcf7*$^{-/-}$ mice and their littermate controls. Significant differences were determined by Wilcoxon test. Bar graphs represent mean taxa abundance ± SEM. * P < 0.05, ** P < 0.01, *** P < 0.001.
